# Importance of TGFβ in Cancer and Nematode Infection and Their Interaction—Opinion

**DOI:** 10.3390/biom12111572

**Published:** 2022-10-26

**Authors:** Marta Maruszewska-Cheruiyot, Michael James Stear, Maja Machcińska, Katarzyna Donskow-Łysoniewska

**Affiliations:** 1 Department of Experimental Immunotherapy, Faculty of Medicine, Lazarski University, 02-662 Warsaw, Poland; 2 Department of Animal, Plant and Soil Science, Agribio, La Trobe University, Bundoora 3086, Australia

**Keywords:** TGFβ, TGFβ mimic, nematode, cancer, immunomodulation

## Abstract

Historically, there has been little interaction between parasitologists and oncologists, although some helminth infections predispose to the development of tumours. In addition, both parasites and tumours need to survive immune attack. Recent research suggests that both tumours and parasites suppress the immune response to increase their chances of survival. They both co-opt the transforming growth factor beta (TGFβ) signalling pathway to modulate the immune response to their benefit. In particular, there is concern that suppression of the immune response by nematodes and their products could enhance susceptibility to tumours in both natural and artificial infections.

## 1. Introduction

The β superfamily of transforming growth factors is a numerous group of evolutionarily conserved ligands involved in the regulation of cellular, physiological, and pathological processes. Signalling by TGFβ influences embryonic development and tissue homeostasis, including angiogenesis, tissue regeneration, modulation of the immune response, extracellular matrix remodelling, cell mobility, and apoptosis in physiological and pathological conditions, especially during development, tumour progression, and metastasis [1,2,3,4,5].

The pleiotropic activity of the signalling pathways induced by TGFβ superfamily factors reflects the complexity of the signal transmitted. This complexity is observed in TGFβ ligands, more than 30 of which have been identified so far. TGFβ occurs in human cells in three isoforms: TGFβ1, TGFβ2, and TGFβ3. Their amino acid sequence similarity is 71–80% [6]. TGFβ isoforms are synthesised as a pre-protein. The native form is dimeric, and the primary structure of active ligands contains a motif of 6 to 12 cysteine residues, referred to as the cystine knot (CK). The presence of the cystine knot is responsible for the formation of homo- and heterodimers of TGFβ factors, and their active form has a molecular weight of approximately 25 kDa.

**Figure 1:**
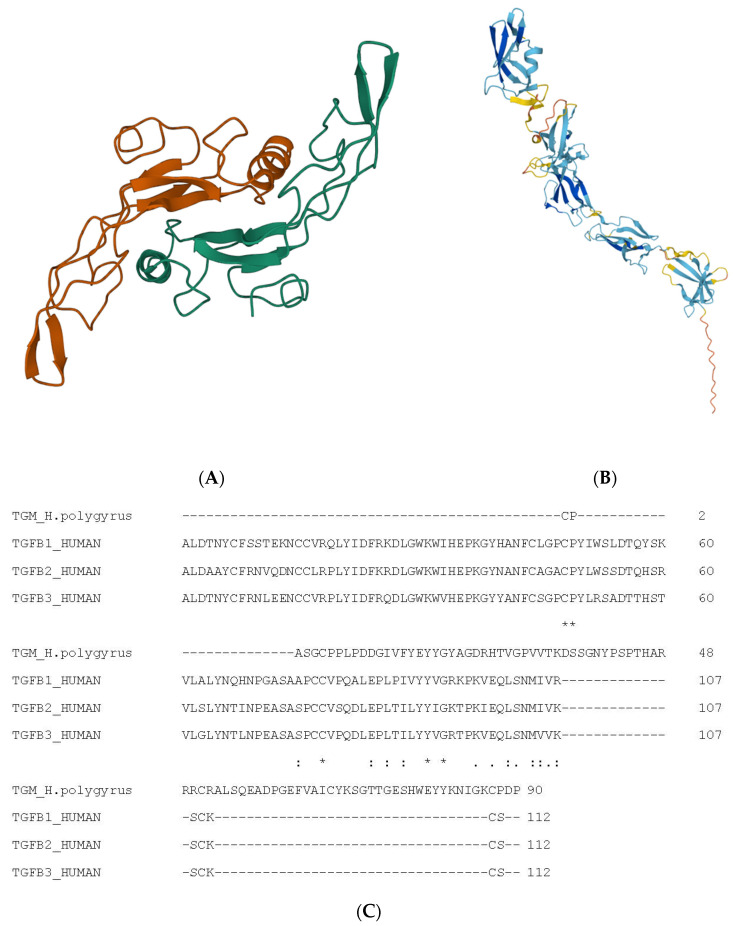
Structure of TGFβ1 and nematode-derived mimic TGFM. (**A**) Structure of TGFβ1. (**A**) Used Mol* to create visualisation [8]. Alpha helices are indicated as coils, whereas beta-pleated strands are indicated as arrows. (**B**) Structure of TGFM. (**B**) Also used Mol* for visualisation. Alpha helices are indicated as coils, whereas beta-pleated strands are indicated as arrows. (**C**) Multiple sequence alignment of TGFβ isoforms and nematode-derived mimic TGFM. The ‘*’ indicates an identical amino acid while the ‘:’ indicates a similar amino acid.

TGFβ1 is synthesised as a 390 amino acid proprotein, which undergoes post-translational processing. Amino acids 1–29 form the signal peptide, amino acids 30–278 form the latency-associated peptide LAP, whereas the remaining amino acids 279–390 form TGFβ1. The LAP and TGFβ1 chains remain noncovalently linked during storage in the extracellular matrix, which keeps TGFβ1 inactive. The sequence of the protein is available in the EBI database (P01137), and the structure (1KLA in the Protein Data Bank in Europe) of the homodimer TGFβ1 (Figure 1A) was determined by Hinck et al. 1996 [7].

Canonical cell signalling induced by ligands belonging to the TGFβ superfamily is mediated by highly specific serine and threonine kinase receptors, which are expressed by most types of human cells. TGFβ receptors are transmembrane glycoproteins with an N-terminal region responsible for ligand binding, a single transmembrane fragment, and a C-terminal cytoplasmic region in which the kinase domain is located. Activation of the canonical TGFβ pathway occurs when the dimeric TGFβ ligand binds to the TGFβ type II receptor dimer, which, having constitutive kinase activity, undergoes autophosphorylation. The TGFβR1 receptor dimer is bound to the TGFβ–TGFβR2 complex, and serine and threonine residues in the repeating glycine and serine residues (GS region) are then phosphorylated by the activated TGFβR2 receptor. The signal is then transmitted through the TGFβ Ina receptors of the Smad cytoplasmic proteins. Canonical signalling of TGFβ is negatively regulated by the Inhibitory Smads (I-Smad) proteins Smad6 and Smad7. The I-Smad proteins, unlike the R-Smad and Co-Smad proteins, do not contain the MH1 domain responsible for DNA sequence recognition and transcriptional activity. The MH2 domain determines the binding of the Smad7 protein to TGFβ receptors, thus competing with the R-Smad proteins. Similarly, TGFβ cell signalling is inhibited by the Smad6 protein, which, by binding to the Smad4 protein, reduces the formation of Smad1–Smad4 complexes [9,10,11,12]. 

### Many Faces of TGFβ

Due to the influence of TGFβ on an array of diverse cellular functions including cell growth, differentiation, adhesion, migration, and apoptosis, perturbations of the TGFβ signalling pathways are involved in the progression of various tumours. TGFβ is a multifunctional cytokine that acts in a cell- and context-dependent manner as a tumour promoter or tumour suppressor. This phenomenon is known as the TGFβ paradox [13]. In healthy cells and early-stage cancer cells, TGFβ ligands stimulate signalling pathways leading to the expression of genes involved in tumour suppression, inhibition of proliferation, stimulation of differentiation, induction of apoptosis or autophagy, elimination of inflammation, and suppression of angiogenesis. In contrast, advanced tumours produce excessive amounts of TGFβ, which contributes to tumour growth, invasion and metastatic spread, and drug resistance [5]. 

The pleiotropic function of TGFβ in tumour development is due to the interaction of this cytokine with various signalling pathways. Activation of the TGFβ type II receptor (TβRII) and, in turn, type I receptor (TβRI) by TGFβ1 transduces signals through receptor-regulated Smads (Smad2/3) and common-partner Smad (Smad4), leading to the transcriptional regulation of target genes. Reflecting its diverse and complex functions in cancer cells, TGFβ upregulates some autophagy-related genes in a Smad4-dependent fashion. Thus, certain hepatocellular carcinoma cell lines undergo cell cycle arrest and apoptosis in response to TGFβ [14].

However, a number of noncanonical TGFβ signalling pathways are responsible for unexpected signalling outcomes or even opposing biological outcomes of TGFβ signalling in the same cells [15]. The activated TGFβ complex transfers signals through molecules such as receptor-associated factor 4 (TRAF4), TRAF6, TGFβ-activated kinase 1 (TAK1), p38 mitogen-activated protein kinase (p38 MAPK), p42/p44 MAPK, phosphoinositide 3-kinase Pi3K/AKT, extracellular signal-regulated kinase (ERK), Rho-like GTPase signalling pathways, JUN N-terminal kinase (JNK), or NF-κB to reinforce or decrease downstream cellular responses [16]. Some of these pathways could be transducers of the TGFβ signal to Smads [17].

Furthermore, the regulation of TGFβ signalling in cells can include the intracellular distribution of the receptors, composition of the receptors, and expression of accessory molecules. For example, mature alternatively activated macrophages (M2) induced by IL-4 require the presence of glucocorticoids (GCs) to express TGFRII on the cell surface to become permissive to the TGFβ [18].

TGFβ is involved in interactions between cancer cells and the host immune system termed as “immunoediting” and summarised in the three “E’s” theory: elimination, equilibrium, and escape. Because of (i) genetic instability and tumour heterogeneity and (ii) immune selection pressure, tumour cells become progressively capable of avoiding immune destruction during carcinogenesis [19,20,21]. Evading immune eradication is a prerequisite for neoplastic progression. The immune escape strategies of cancer may be classified into two main mechanisms. First, cancer cells may become invisible to the immune system. This can be achieved by losing or downregulating MHC and/or molecules involved in antigen presentation, thereby preventing their recognition by the immune system. Second, cells may “defend” themselves to become resistant to immune eradication. This can be achieved in several ways: by becoming resistant to apoptosis, expressing inhibitory ligands that deactivate immune cells, and/or inducing an immunosuppressive microenvironment, the TME [19].

In cancer, TGFβ is recognised as one of the most important regulators in the tumour microenvironment (TME), which is a highly heterogeneous milieu consisting of different cell types. The TME includes the presence of immunosuppressive cell populations such as tumour-infiltrating myeloid cells, including myeloid-derived suppressor cells (MDSCs) and M2-like tumour-associated macrophages (TAMs). Likewise, the presence of immune regulatory populations, including regulatory T cells (Tregs), regulatory B cells (Bregs), and regulatory dendritic cells (DCregs), can mediate immunosuppression [22]. These cell types beside cancer cells are not just sources of TGFβs; they seem to exploit autocrine or paracrine TGFβ for their expansion, polarisation, and behaviour towards a tumour-promoting role rather than tumour elimination [22]. Tumour immune escape is complex, and a crucial aspect is promoting the expansion and activation of immature DCs. The immature DCs that uptake apoptotic and necrotic DCs convert into tolerogenic DCs (tDCs) with enhanced TGFβ secretion [23]. tDCs display low expression of costimulatory molecules such as CD80 and CD86 as immature DCs; but they simultaneously have high expression of inhibitory molecules (TRAIL, PD-L1, DC-SIGN, and CTLA-4) and immunosuppressive molecules (e.g., TGFβ, IL-10, IL-27, NO, and IDO) and, in turn, support the Treg differentiation of type 1 Tregs (Tr1) in response to IDO, IL-10, and TGFβ. 

Naïve CD4^+^ T cells differentiate into CD25^+^ Foxp3^+^ Tregs (Tregs) in the thymus and into CD25^−^ Tregs including IL-10^+^ Tr1, TGFβ^+^ Th3, and Foxp3^+^ cells (iTregs) in the periphery. Treg cells promote tumour growth and progression through multiple inhibitory pathways, and TGFβ is secreted at high levels by Treg cells in the tumour microenvironment. Blockade of TGFβ expressed on the surface of Treg cells using neutralising antibodies improved immunity to melanoma and suppressed the metastasis of pancreatic tumours in mice [24,25]. Furthermore, TGFβ mediates T-cell suppression via programmed death-1 (PD-1) coinhibitory receptor 1 (immune checkpoint) upregulation through Smad3-dependent and Smad2-independent transcriptional activation in T cells and in the TME [26].

In response to the TGFβ released by different cell types, including tumour cells in the TME, circulating monocytes that express high numbers of TGF- receptors migrate into the TME, where they differentiate into tumour-associated macrophages (TAMs). On these cells, TGFβ induces markers specific for M2-type macrophages with tumour supportive function [13].

Other immune cells such as cytotoxic T cells (CTLs), natural killer (NK), and neutrophils involved in the anticancer response are also regulated by TGFβ. In the mouse system, TGFβ prevents the activity of cytotoxic CD8^+^ T cells by inhibition of perforin, granzymes (GzmA, GzmB), interferon-γ (IFN-γ), and FasL expression [27]. Correct NK signal transduction by NKG2D surface receptor–ligand binding culminates in the degranulation of NK cells to eliminate tumour cells. NKG2D ligands on tumour cells are downregulated, among other molecules, by TGFβ to escape NK-cell-mediated immune surveillance [28]. TGFβ can also inhibit NK cell activity by decreasing IFN-γ production by these cells [29].

Neutrophils are the main group of cells that infiltrate tumours, and they have a critical function in the immunosuppression of the TME as the tumour evolves. Similarly to M2, N2 neutrophils (N2 TANs) display protumourigenic activity, and the TGFβ within the tumour microenvironment induces N2 cells. TGFβ-stimulated N2 secretes different molecules that shape the TME. Neutrophils can be activated to display a stronger antitumour phenotype by blocking TGFβ [30,31].

The epithelial–mesenchymal transition (EMT) plays a significant role in metastasis with a process crucial to the process of tumour cell dissemination [32]. Epithelial cells lose their polarity during EMT when cell adhesion structures such as gap junctions are eliminated. E-cadherin and other cell adhesion molecules are downregulated, and N-cadherin takes their place, allowing for more transitory cell-to-cell and cell–matrix adhesions [33]. Vimentin is a protein that is highly expressed in mesenchymal cells and is linked to enhanced metastasis [34]. Signalling by TGFβ is critical in the EMT. TGFβ modulates Snail activity, one of several transcription factors involved in the EMT; hence, loss of epithelial markers and rise of mesenchymal markers influence invasion and metastasis [35].

TGFβ is a key molecule that initiates a pathway with independent and complementary immunosuppressive functions, and a high level of blood TGFβ is associated with a poor cancer prognosis. Therefore, it is not surprising that recent reports have shown that upregulation of TGFβ signalling was a major driver of targeted and conventional therapy resistance in a variety of solid tumours [36]. Despite promising clinical activity, only a minority of patients respond to anti–PD-1/PD-L1 therapies with checkpoint inhibitors (CPIs). TGFβ has been associated with resistance to immune checkpoint blockade. The concept of PD-L1/TGFβ dual inhibition was to simultaneously target a receptor (PD-L1) and TGFβ to block tumour-cell-intrinsic and -extrinsic immunosuppressive pathways, respectively. 

Indeed, in PD-L1/TGFβ dual inhibition, increased infiltration of CD8 T cells, NK cells, dendritic cells, and M1 macrophages was seen in responders [37]. The combined inhibition of TGFβ and PD-1/PD-L1 signalling may act in synergy to elicit antitumour responses and prevent metastasis as shown in various tumour models. Blocking the PD-L1/PD-1 axis and TGFβ actions reduced TGFβ signalling in stromal cells, facilitated T-cell penetration into the centre of the tumour, and provoked vigorous antitumour immunity and tumour regression in mice. Inhibition can act by suppressing Tregs and inhibiting the cancer-associated epithelial–mesenchymal transition [38]. 

## 2. TGFβ Signalling in Helminths

Even such primitive animals as sponges and *Trichoplax* produce molecules belonging to the TGFβ family [39]. However, TGFβ seems to be unique to the animal kingdom. There are no documented members of the protein family in other kingdoms of living organisms. Several species of nematodes, both free-living and parasitic, have TGFβ signalling pathway components.

The best-described TGFβ pathway in nematodes is TGFβ signalling in *Caenorabditis elegans*. The TGFβ pathway plays fundamental roles in the development of this free-living roundworm. Five ligands (Ce-DBL-1, Ce-DAF-7, CeUNC-129, Ce-TIG-2, and Ce-TIG-3) and three receptors (Ce-DAF-1, Ce-DAF-4, and Ce-SMA-6) have been identified, and two signal pathways have been described for *C. elegans*. 

The first pathway controls dauer larvae. Dauer larvae develop in response to unfavourable environmental conditions. Ce-DAF-7 influences entry and exit from the dauer stage and is a ligand for Ce-DAF-1 (type I) and Ce-DAF-4 (type II) receptors. Ce-DAF-8 and Ce-DAF-14 are components involved in signal transmission, whereas Ce-DAF-3 antagonistically acts towards them [40,41]. The dauer form in free-living roundworms is hypothesised to be equivalent to the infective L3 stage of parasitic nematodes [42,43,44]. It appears to be critical in the evolution of parasitism. 

The second TGFβ-related pathway, called Sma/Mab, is involved in body size control, regulation of gland cell morphology, development of mail tail, immune defence, mesoderm differentiation, and reproductive ageing [41,45,46]. Ce-DBL-1 is a ligand for Ce-SMA-6 and Ce-DAF-4 receptors in this pathway. SMA-2, SMA-3, and SMA-4 have been identified as Smad components and are involved in the Sma/Mab signal pathway [41]. 

DAF-7, DAF-1, and DAF-4 are strongly conserved throughout nematode phyla. However, the regulators of the TGFβ pathway in *C. elegans* have also been found in Clades IV and V (DAF-8 and DAF-3) or in Clade V (DAF-14) only [47]. Parasitic nematodes independently evolved at least 15 times from free-living Nematoda [48]. However, the role of the TGF pathway in the evolutionary origin of parasitism is unclear. One hypothesis states that the appearance of TGFβ in the evolution of nematodes had a key role in dauer formation and the emergence of invasive larvae from this stage [49]. An alternative hypothesis considers TGF signalling to be primarily in development from iL3 to adult [47].

Two TGF-related genes have been found in *Brugia malayi.* This nematode is transmitted by mosquitoes and causes lymphatic filariasis in humans. Larval stages are found in the blood, whereas adults settle in lymphatic vessels. A transforming growth factor homolog-1 (Bm-tgh-1) shows similarity to genes encoding proteins in the dpp/Dbl-1 family. Bm-tgh-1 is mostly expressed during parasite growth when nematodes are present in the host [50]. A second molecule, Bm-TGH-2 displays similarity to DAF-2 from *C. elegans* and to human TGFβ. The similarity between Bm-TGH-2 and human TGFβ is restricted to the C-terminal domain with 38% identify. The gene encoding Bm-TGH-2 is maximally expressed in the microfilarial stages. As the parasite is exposed to various immune factors in the host blood, Bm-TGH-2 may play a significant role in modulating the immune response. Tgh-2 is expressed during the adult stage, in male as well as female species of *B. malayi.* Recombinant Bm-TGH-2 binds to the TGFβ receptor, and this binding can be partially inhibited by human TGFβ [51]. 

*Brugia pahangi* has a gene encoding a TGFβ receptor homologue termed Bp-trk-1 [52]. Bp-trk-1 shows similarity to TGFβ type I receptors and SMA-6 from *C. elegans* [53]. Furthermore, filariae may be rich in molecules with similar structures to TGFβ. Antibodies that recognise the latent form of human TGFβ react with *B. malayi* as well as many filarial worms including *Onchocerca volvulus*, *Onchocerca gibsoni*, *Onchocerca ochengi*, *Onchocerca armillata*, *Onchocerca fasciata*, *Onchocerca flexuosa*, *Wuchereria bancrofti*, and *Dirofilaria* sp. These antibodies may detect molecules with structural similarity to TGFβ1 [54].

Furthermore, three proteins in the hookworm *Ancylostoma caninum* have been identified as TGF family members. *A. caninum* develops in dogs and cats. Humans can also be infected with *A. caninum* as an accidental host. *A. caninum* DBL-1 (Ac-DBL-1) is involved in parasite growth regulation, and the protein has a similar amino acid sequence to *C. elegans* DBL-1. The highest expression of Ac-dbl-1 is observed in the adult male stage; hence, DBL-1 from *A. caninum* probably performs the same function as in *C. elegans*—mail tail growth regulation. Another one, with the highest similarity to *C. elegans* DAF-7, is called Ac-DAF-7 and regulates arrested development. Tissue-arrested L3 and reactivated L3 stages of *A. caninum* are characterised by the highest Ac-daf-7 expression [55]. In addition, human TGFβ can reactivate the tissue-arrested form of *A. caninum* but cannot induce the development of environmental L3. It is possible that L3 possesses receptors for TGF molecules of mammalian origin [56]. 

*Haemonchus contortus* is an important parasite found in the abomasum (true stomach) of goats and sheep. Two receptors belonging to the TGFβ family have been identified for *H. contortus.* One has domains characteristic of a TGFβ type I: receptor Hc-TGFBR1, and one is a TGFβ type II receptor: Hc-TGFBR2 [57,58]. Both of them play a role in developmental processes of *H. contortus*. Hc-TGFBR1 gene expression is observed during all developmental stages of the parasite. Interestingly, Hc-TGFBR1 is structurally more similar to human TGFBR1 than to DAF-1 from *C. elegans*, suggesting a more similar function to a molecule of human origin [57]. Furthermore, Galunisertib, a TGF-type I receptor inhibitor utilised in clinical trials involving cancer patients [59], can block the transition of free-living L3 into the parasitic L4 of *H. contortus* [55,57]. Hc-tgbr2 is expressed in all stages of the parasite, with the greatest levels of expression found in infective L3 and adult male species. Silencing of the gene reduced the level of the L3 to L4 transition in vitro [56]. In addition, two TGF signalling ligands for *H. contortus* have been characterised. *H. contortus* DAF-3 (Hc-DAF-3) shows similarity to DAF-3 from *C. elegans* and is classified as Co-Smad protein. Hc-daf-3 is expressed at the highest level by L3 and adult female species [60]. Hc-TGH-2 is the second ligand described for *H. contortus*. The Hc-tgh-2 gene is expressed by all developmental stages, with iL3 expressing the most. Hc-TGH-2 seems to play an important role in the transition from the free-living to parasitic stage, as well as in digestion, absorption, and reproductive development, based on the localisation of the ligand in parasite body [61].

Molecules with similar sequences to TGF ligands have also been found in *Heligmosomoides polygyrus*, *Necator brasilienisis*, and *Teladorsargia circumcincta*, although their functions have not been studied. The TGH-2 subfamily showed similarity to Ce-DAF-7, TGH-2 from *B. malayi* and mammalian TGFβ. Interestingly, tgh-2 expression varies in four different Trichostrongyloid nematode parasites. The maximum expression occurs in adults of *H. polygyrus* and *T. circumcincta*, but only in L3 in *H. contortus* and *N. brasiliensis* [62].

Despite the evolutionary distance between nematodes and other helminths, such as flukes and tapeworms, TGF family factors have been identified in all groups. In the parasitic flatworm *Schistosoma mansoni,* a parasite of humans, many molecules classified as members of the TGFβ family have been identified including receptors SmRK1 [63] and SmRK2 [64], Smad signalling components [65,66,67], and ligands: an Inhibin/activin-like molecule (SmInAct) [68] and BMP-like molecule (SmBMP) [69]. All of these molecules are engaged in a variety of activities that require TGF signalling, such as parasite development, fluke adult male and female interactions, and host–parasite interactions [70]. Interestingly, the SmRK1 receptor responds to human TGFβ1 and TGFβ3, indicating that host ligands could participate in parasite development [71]. The TGF-like ligand SmBMP was recognised as an excretory–secretory product of adult male species; hence, it can affect host tissues by direct contact. Because SmInAct is not secreted by *S. mansoni*, it is unlikely to be involved in TGF signalling in humans infected with the parasite. The main function of SmInAct seems to be the regulation of parasite reproduction [68].

TGF-like molecules have also been found in two tapeworm genera. In *Taenia solium*, receptors and Smad-like elements have been identified. In vivo tests showed that TGFβ supports the survival of *T. solium* cysticerci as well as the development and reproduction of *Taenia crassiceps* cysticerci [72]. In the human parasite *Echinococcus multilocularis,* EmTR1, a member of the TGFβ type I receptor family, has been identified. EmTR1 binds host origin BMP2 and may influence parasite development [73]. A number of Smad family members have also been identified for *E. multilocularis* [73,74,75]. One of them, EmSmadE is preferentially phosphorylated by human TGFβ type I and the BMP type I receptors [75]. Smad homologues have also been discovered in *E. granulosus*. One of them, EgSmadE can be translocated into mammalian cells’ nucleus in the presence of human TGFβ 1 and BMP2, suggesting that mammalian ligands can activate a TGFβ-like pathway in helminths [76]. Host-derived growth factors and cytokines, such as TGFβ, may play a role in helminth growth and development during infection. Studies on mouse models infected with nematodes indicate a significant effect of TGFβ on the success of infection. The presence of TGFβ in the host’s organism results in the increased survival of nematodes; hence, TGFβ signalling alternation results in greater resistance to the parasite [77,78].

Most nematode research has concentrated on the TGF pathway’s developmental role. However, TGFβ can influence nematode development and other processes occurring in the host and parasite. An intriguing but unconfirmed hypothesis is that parasites may have changed genes involved in endogenous body architecture to engage with their host’s immune system [51].

### Nematode TGFβ Mimic

Compounds that have a different structure but function similarly to TGF ligands have been reported in parasitic nematodes. *Heligmosomoides polygyrus* is among the best understood of all nematode infections, and immune modulation has been clearly demonstrated [79]. *H. polygyrus* has a direct life cycle [79]. Adults live in the small intestine where they breed; eggs are laid by adult female species and excreted in the faeces. They develop through two moults into infective third-stage larvae. Infective larvae are ingested in natural infections but usually orally gavaged in experimental infections. Within 24 h, larvae have penetrated into the submucosa where they develop over the next ten days and undergo two moults before emerging into the lumen of the small intestine. About two weeks after infection, adult female species produce eggs that can be seen in the faeces. 

**Figure 2:**
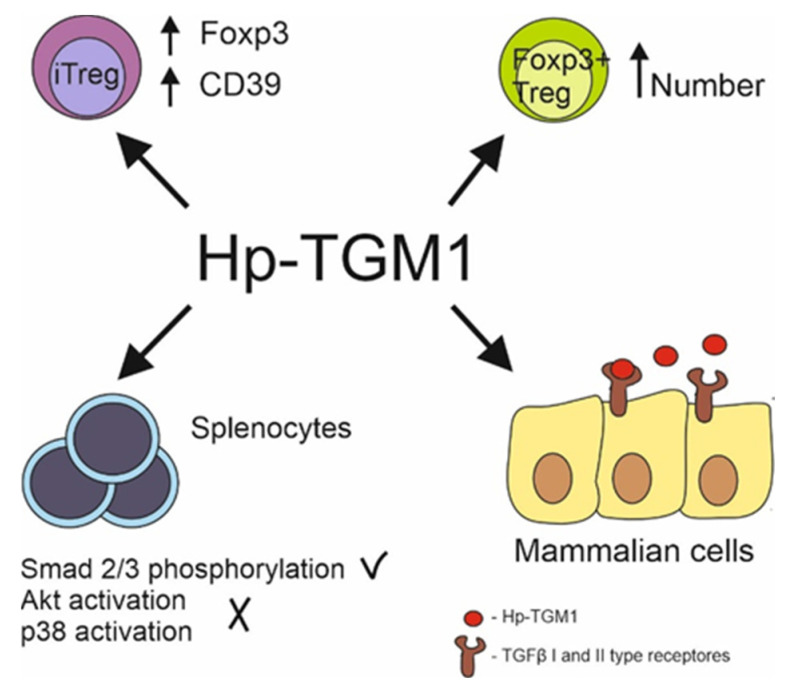
Hp-TGM1 functions similarly to mammalian TGFβ despite lack of homologies between proteins. Hp-TGM1 binds to mammalian TGFβ I and II type receptors; induces Smad 2/3 phosphorylation with lack in Akt and p38 signal pathway activation in murine splenocytes; induces generation of mice and human Foxp3^+^ Treg cells; increases Foxp3 and CD39 expression in induced Treg same as mammalian TGFβ.

*H. polygyrus* produces at least five molecules that modulate immune responses [80], and these influence the immune response at several levels. One of them is a TGFβ mimic (*H. polygyrus bakeri* TGFβ mimic, Hp-TGM), which influences the production of regulatory T cells. A family of ten Hp-TGMs are members of the complement control protein (CCP) superfamily, which also contains HpARI (*H. polygyrus bakeri* alarmin release inhibitor) and HpBARI (*H. polygyrus bakeri* binds alarmin receptor and inhibits). Hp-TGM has high immunomodulatory potential [81] and consists of five atypical domains. The Hp-TGM family has no homology with mammalian TGFβ or other members of the TGFβ family [82]. However, Hp-TGM can bind to TGFβ receptors and possesses TβRI and TβRII receptor-binding sites. Moreover, HP-TGM competes with mammalian TGFβ for TGFβ receptors [83]. Although the TGM D3 domain and TGFβ bind the same residues in the TGFβ receptors, there is little sequence similarity between the molecules (Figure 2).

The family members that exhibit functional activity similar to TGFβ have a similar or identical sequence to Hp-TGM in the first three CCP domains, whereas domains 4 and 5 were more variable. This suggests that domains 1–3 are necessary for function [82]. 

Hp-TGMs 1–5 occur in adult nematodes, whereas 7–10 are produced by the L4 stage of the parasite. Five Hp-TGMs have been tested, and two of them were functionally active in an MFB-11 reporter assay [82]. Hp-TGM1 mimics the biological and functional properties of TGFβ1. Both TGFβ1 and Hp-TGM1 directly signal through the same combination of type I and II receptors on mammalian cells. Tests on murine splenocytes showed that Hp-TGM1 induced the phosphorylation of Smad2/3 with no activation of Akt and p38 pathways, which is similar to TGFβ1. Furthermore, Hp-TGM1 induces mouse and human Foxp3^+^ Treg cells [81]. 

In contrast to TGFβ1, TGM is synthesised as a 420 amino acid polypeptide and does not undergo post-translational processing. It contains five tandem CCP domains labelled D1–D5 (Figure 1B). D3 competes with TGFβ for the receptor TβRII [83]. The structure of domain 3 has been determined [82] but not the structure of the whole molecule. Figure 1B is a predicted structure (https://alphafold.ebi.ac.uk/entry/A0A2D1LW19 accessed on 19th August 2022). Figure 1C shows a multiple sequence alignment performed with the Clustal omega of TGFβ1, TGFβ2, TGFβ3, and TGM-D3. TGM-D3 was derived from the PDB file 7SXB, and it differs from the corresponding sequence of TGM (A0A2D1LW19) in the first four amino acids: CPAS in 7SXB but GSGT in A0A2D1LW19. The multiple sequence alignment shows that only seven amino acids are conserved between TGM and the three TGFβ sequences, although four of these seven amino acids are cysteines.

Additionally, a comparative study of Hp-TGM1 with mammalian TGFβ showed that the parasite molecule is a more effective Treg inducer. Stimulation of mouse T cells with Hp-TGM1 significantly increases Foxp3 and CD39 expression levels in induced regulatory T cells (iTregs), despite slower signal induction [84]. Hp-TGM properties are presented in Figure 2.

Hp-TGM1-induced Tregs are able to limit the progression of autoimmune disease in mice models, demonstrating strong suppressive activity. In the experimental autoimmune encephalomyelitis (EAE) model of multiple sclerosis, iTreg under the influence of Hp-TGM1 inhibited the autoantigen-specific Th17 response. In the dextran sulphate sodium (DSS) colitis model, HP-TGM1-iTreg was more stable in vivo as a result of greater retention of Foxp3 expression and lower conversion to a ROR-γt^+^ phenotype [85]. 

Treatment with recombinant Hp-TGM increased the number of Tregs in draining lymph nodes at the site of the graft transplant in mice, resulting in delayed allograft rejection [81]. Hp-TGM decreased allergic eosinophilia in diverse airway inflammatory mouse models by impacting on epithelial-derived cytokines and IL-4 responses [86]. In addition, we observed that Hp-TGMs are produced by the *H. polygyrus* L4 stage at a higher level in culture in the presence of the opposite sex in comparison with single-sex cultures. This indicates the importance of this group of proteins for the survival and reproduction of the parasite [87]. 

Both parasites and tumours need to suppress the immune response to increase their chances of survival. They can both use the TGFβ signalling pathway to modulate the immune response to their benefit. Furthermore, mammalian ligands can activate a TGFβ-like pathway in helminths, and parasitic TGFR ligands can influence the host TGFβ pathway. 

Consequently, suppression of the immune response by nematodes and their TGFR ligands can affect tumours in both natural and artificial infections, although the effect of the parasitic origin TGFβ and TGM in cancer cells has not been studied. At this point, deeper collaboration and interaction between parasitologists and oncologists become necessary. 

## 3. Potential Influence of Nematode Infection on Tumour Development

Parasitic nematodes pose significant problems in human and animal medicine. Helminth infections in developing areas of the world are still very common [88,89]. About 24% of humans are infected by gastrointestinal nematodes [89]. Hundreds of millions of people are infected with roundworms, including hookworms and whipworms [90]. Nematode infections of livestock cause economic losses all over the world. Despite widely available antihelminthic drugs, resistance of nematodes to the drugs is increasingly apparent [91]; hence, parasitic infection affects human and animal health with knock-on effects on the economy. 

Nematodes show amazing immunomodulatory potential. Some helminths inhibit the immune response by activating regulatory T cells, either through the expansion of the pre-existing Tregs or through inducing de novo differentiation of peripheral T cells into the Treg subset [92]. Higher levels of IL-10 and TGFβ secretion by Treg suppress Th1 and Th2 cell activity, consequently protecting both the parasite against expulsion and the host against damage caused by inflammatory reactions [93]. 

Consequently, nematodes and their products are promising treatments for autoimmune diseases and allergies [94,95]. Autoimmune diseases are an increasing medical problem in modern societies. Immune system intolerance to self-antigens is noted in 5% of the population of developed countries [96]. To date, no effective treatment of autoimmune diseases has been developed. Clinical trials of helminth therapy in patients suffering from ulcerative colitis, Crohn’s disease, or multiple sclerosis have shown promising results. As part of the treatment, patients are given the nematodes *Trichuris suis* or *Necator americanus* [97,98,99,100]. There are still many questions to be answered. The influence of parasitic infection on patients with abnormal immune responses, especially over a longer period of time, has still to be rigorously evaluated. It is particularly important as patients with autoimmune diseases are more likely to develop cancer as a result of underlying immune system dysregulation or treatment with immunosuppressants such as corticosteroids. Patients with inflammatory bowel disease are at an increased risk of colonic epithelial dysplasia and carcinoma, that is, at least three times greater than that in the general population [101]. Patients with multiple sclerosis are more likely than healthy persons to develop a variety of cancers [102]. The inflammatory environment during colitis affects nematode survival, immunomodulatory properties, and immunogenicity in a mice model [103,104]. During helminth therapy or normal infection, the host is exposed to the full spectrum of helminth-derived agents. Other studies have focused on single parasite-derived proteins [105,106]. Ruling out their carcinogenicity is crucial, especially as some parasitic infections have been closely associated with tumours, either predisposing to or protecting against. Infectious diseases have been associated with 16.1% of cancers [107]. Unfortunately, not all the links are definitively proven. Most epidemiological observations are not supported by experimental models.

A number of flukes and tapeworms, including *Schistosoma haematobium*, *Schistosoma japonicum*, *Schistosoma mansoni*, *Spiromera mansonoides*, *Opisthorchis viverrini*, *Clonorchis sinensis*, *Taenia formis*, and *Taenia solium*, have significant tumour-promoting activity [108,109]. Products from the small intestinal nematodes *Trichostrongylus vitrinus*, *Trichostrongylus colubriformis*, *Cooperia curticei*, and *Nematodirus battus* and the abomasal nematode *Teladorsagia circumcincta* have all been shown to produce overproliferation in normal intestinal epithelial cells and/or cell lines [110]. Exposure to the fish parasite Anisakis is a risk factor for the development of stomach or colon adenocarcinoma in humans. A serological study indicated that the ratio of positivity to specific IgE, IgA1, and IgG1 against the *Anisakis* recombinant antigens was significantly higher in cancer patients than in controls [111]. 

Nematode infection often produces a protective immune response that controls but does not completely eliminate or prevent infection [112]. Nematodes produce a variety of molecules to enhance and suppress the immune response [112,113,114]. An excellent example is the multihost nematode *Trichinella spiralis*, which possesses antitumour and protumour activities [115]. 

Some helminth components might enhance the immune response and so abort the development of some cancers. For example, infection with the nematode *Nippostrongylus brasiliensis* can systemically alter genital tract mesenchymal markers in a way that may impair cervical cancer cell progression induced by human papillomavirus (HPV). *N. brasiliensis* exposure significantly altered the epithelial–mesenchymal transition (EMT) marker expression while decreasing HPV type 16 (HPV16) pseudovirion internalisation [116]. Although the study does not indicate the molecule responsible for the effects, these findings reveal the anticancer property of helminth molecules. 

The excretory–secretory (ES) products of *H. contortus* are involved in histopathological changes observed in the parasitised abomasum, ES-induced extensive vacuolation with reduced numbers of adherent HeLa cells [117]. 

Furthermore, in Hepa1-6 mouse hepatoma models, the ES of the nematode *Angiostrongylus cantonensis* significantly reduced tumour growth, and the effect was correlated with significantly higher CD3^+^, CD4^+^, and CD8^+^ T-cell counts. The antitumour effect was confirmed in vitro. The ES significantly accelerated human hepatocarcinoma HepG2 cell apoptosis [118]. 

Proapoptotic activity through a mitochondrial pathway was also indicated for the ES of *T. spiralis* muscle larva (ML). In small-cell lung cancer cells, H446 co-cultured with the ES indicated increased the expression of pro-apoptosis genes Bax, Cyt-C, Apaf-1, caspase-9, and caspase-3 and decreased the expression of anti-apoptosis genes Bcl-2 and Livin [119]. 

Nematodes and their products have been found to trigger apoptosis pathways and anergy in host immune cells including immune cells and intestinal epithelial cells. Depending on the target, nematodes can upregulate and downregulate apoptosis-related genes and create a suitable environment for development [120].

On the other hand, while acute inflammation is a part of the defence response, chronic inflammatory lesions, such as those induced by persistent parasite infections, are associated with increased cancer risk. Inflammation plays a decisive role in the initiation, promotion, progression, metastasis, and resistance to antitumour therapies. The best example of such an interaction is *Schistosoma haematobium* fluke infection. *S. haematobium* is endemic in most countries in Africa and the eastern Mediterranean region. The parasite is classified as a group 1 biological carcinogen and is a cause of bladder cancer as a long-term sequela of chronic infection and inflammation [121]. Adult worms can live in human tissues for more than 30 years. Eggs are deposited in the terminal venules of the bladder where they mature to schistosomula, a developmental stage that releases proteolytic enzymes that facilitate larval movement through the tissues into the genitourinary tract. During infection, eggs are deposited in the mucosa and submucosa of the bladder and lower ureters. Antigens from *Schistosoma haematobium* have been shown to induce urothelial dysplasia and inflammation, whereas the eggs cause physical irritation and inflammation, which leads to squamous cell carcinoma (SCC) of the urinary bladder [122]. There is limited information on nematode-induced cancer as a consequence of inflammation. In the early phase of colitis-associated colon cancer (CAC), before DSS-induced inflammation, *H. polygyrus* infection accelerates the inflammatory immune response in the colon, which enhances tumour development [123].

Over 30 suppressor molecules have been identified from different nematode species [79]. These molecules fall into three main categories: those that reduce the intensity of the Th2 response, those that inhibit protective mechanisms, and those that suppress existing immune responses such as mimics of TGFβ. *H. polygyrus, B. malayi, Strongyloides stercoralis, Strongyloides ratti, Parastrongyloides trichosuri, A. caninum, T. circumcincta, Nipostrongylus brasiliensis*, and *H. contortus* all influence TGFβ signalling [124].

Recently, we confirmed that intestinal nematode infection enhanced the concentration of TGFβ in mouse serum. This elevation was associated with different forms of metastatic foci in the lungs. Although, nematode infection does not play an essential role in the induction of host tolerance to EL4 lymphoma metastasis, in mice experimentally injected with EL4 lymphoma cells, a lower frequency of lung metastases was observed in mice previously infected with *H. polygyrus*, suggesting that pre-infection of mice with nematodes renders them resistant to tumour development in lungs. In pre-infected mice, tumour nodules were found growing in the mesenteric lymph nodes (MLNs). Although a lower number of lung nodules were detected, these were of larger size than those of the EL4 control mice [125]. In a separate study, *H. polygyrus* antigen reduced murine and human colorectal cancer cell (CRC) proliferation, which was associated with the increased expression of p53 and p21. Furthermore, those products increased murine CRC cell migration [126]. Although the direct influence of Hp-TGM on metastasis has not been demonstrated so far, it cannot be ruled out that this molecule is responsible for at least part of the observed effect of *H. polygyrus*. Developments in the fields of cancer research and host–parasite interactions in the last decades have led to a better understanding of the appropriate mechanisms. Although TGFβ-mediated immunosuppression during nematode infection and in tumours is important, the relationship between them is still poorly understood.

## 4. Conclusions

Helminth infections are well-documented as providing some benefit to the host by limiting autoimmune and allergic symptoms in infected individuals partly by influencing TGFβ signalling. Molecules with similar sequences to host TGF ligands have also been found in nematodes. TGFβ is overproduced by various cells during both parasitic infections and cancer (Table 1). TGFβ signalling acts as a negative regulator of antitumour immunity. 

Although the mechanisms of action triggered by the nematodes and some of their products involved in modulating cancer development are diverse and not yet fully described, the cooperation between TGFR ligands of the host and nematode can influence the final outcome of cancer.

Autoimmune disease patients are at an increased risk of a variety of cancers. Therefore, the mechanisms of helminths-mediated immune modulation should be precisely defined before nematodes or their products are used for treatment in autoimmune diseases. It is critical to understand the complex mechanism of TGF pathways to better predict if the combinations of the host and parasite TGF will influence cancer development.

## Figures and Tables

**Table 1:** Comparison of TGFβ participation in various processes during helminthic infection and advanced cancer progression.

Process	Helminths	Cancer
**Signalling** **modification**	Increased level of TGFβ and modification of molecules involved in TGFβ signalling pathway levels: - Induction of TGFβ production by host cells; - Modification of host ligands and receptor production; - Secretion of TGFβ signalling molecule homologs and mimics by parasite.	Excessive production of TGFβ by cancer cells under genetic alternations.
**Growth**	Influence on helminth life cycle: - Parasite growth induction; - Helminth development regulation.	Increased tumour growth: - Proliferation; - Autophagy; - Apoptosis.
**Propagation and** **spreading**	Influence on parasite survival and reproduction: - Decreased resistance to infection.	Increased invasion and metastasis: - Extracellular matrix (ECM) remodelling; - Epithelial–mesenchymal transition (EMT).
**Immunosuppression**	Creation of a milieu conducive to the protection of the helminth against the antiparasitic immune response: - Treg cells generation and expansion; - Th1 and Th2 cell activity suppression.	Creation of immunosuppressive tumour microenvironment: - Expansion of Tregs; - Promotion of tumour-associated macrophages (TAM); - Inhibition of Th1 and cytotoxic T cells, antigen-presenting cells (APC), NK cells, and neutrophile generation and activity.

## Data Availability

Not applicable.
